# Mobile sexual health services for adolescents: investigating the acceptability of youth-directed mobile clinic services in Cape Town, South Africa

**DOI:** 10.1186/s12913-019-4423-4

**Published:** 2019-08-19

**Authors:** Philip Smith, Tsidiso Tolla, Rebecca Marcus, Linda-Gail Bekker

**Affiliations:** The Desmond Tutu HIV Centre, Institute for Infectious Disease and Molecular Medicine, Faculty of Health Science, University of Cape Town, Anzio Road, Observatory, Cape Town, 7925 South Africa

**Keywords:** Acceptability, Mobile clinic, Epidemiology, Usability, HIV, Differentiated healthcare

## Abstract

**Background:**

The Human Immunodeficiency Virus (HIV) epidemic is growing rapidly among South African adolescents and young adults (AYA). Although HIV counselling and testing, HIV prevention and treatment options are widely available, many AYA delay health-seeking until illness occurs, demonstrating a need for youth responsive, integrated sexual and reproductive health services (SRHS). While feasibility and cost-effectiveness have been evaluated, acceptability of mobile clinics among AYA has yet to be established. The objective of this study was to investigate patient acceptability of mobile AYA SRHS and compare mobile clinic usage and HIV outcomes with nearby conventional clinics.

**Methods:**

Patients presenting to a mobile clinic in Cape Town were invited to participate in an acceptability study of a mobile clinic after using the service. A trained researcher administered an acceptability questionnaire. Mobile clinic medical records during the study period were compared with the records of AYA attending four clinics in the same community.

**Results:**

Three hundred three enrolled participants (16–24 years, 246 (81.2%) female) rated mobile AYA SRHS acceptability highly (median = 4,6 out of 5), with 90% rating their experience as better or much better than conventional clinics. The mobile clinic, compared to conventional clinics, attracted more men (26% v 13%, *p* < 0,000), younger patients (18 v 19 years, *p* < 0,000), and yielded more HIV diagnoses (4% v 2%, *p* < 0,000).

**Conclusions:**

Given the high ratings of acceptability, and the preference for mobile clinics over conventional primary health clinics, the scalability of mobile clinics should be investigated as part of a multipronged approach to improve the uptake of SRHS diagnostic, prevention and treatment options for AYA.

**Electronic supplementary material:**

The online version of this article (10.1186/s12913-019-4423-4) contains supplementary material, which is available to authorized users.

## Background

Adolescents and young adults (AYA, 10–24 years) commonly express a desire to find out more about sex and sexual health, but tend to avoid sexual and reproductive health services (SRHS) because they experience real and perceived access barriers [1–6] . Barriers regularly cited involve anticipation of embarrassment, fear of loss of privacy, and fear of physical examination [6]. Although some AYA may visit pharmacies and general practitioners, these alternative services may be out of reach because they are practically inaccessible due to cost, far traveling distance or the long time required for visits [7]. Many young South Africans only enter healthcare once ill and in need of care [6, 8, 9].

The unique psychosocial and biological transitions place AYA in high disease burden communities at significant risk of human immunodeficiency virus (HIV) acquisition [1, 8, 10, 11]. Sexual debut [12, 13] and developing sexuality both occur during this developmental phase and frequently co-occur with sexually transmitted infections (STIs) [3, 7, 14] and unintended pregnancies [15–17]. While HIV incidence and related mortality have declined in recent years [18], South African AYA have not experienced the same declines [3, 19–21].

Despite the recommendation to improve HIV testing in key populations [22], many AYA living with HIV remain unidentified until symptomatic [9, 23–25]. The global scale-up of antiretroviral treatment (ART) has dramatically improved the quality of life for many people living with HIV. In combination with biochemical prevention methods, such as pre-exposure prophylaxis (PrEP), access to ART programs can significantly curb the HIV epidemic. Attaining this ambitious goal requires innovation that links young South Africans to SRHS, including diagnosis, prevention, treatment, adherence and viral suppression [1, 26–29]. With this in mind, youth-friendly services have been recommended for AYA [6, 30–32]. Youth-friendly services typically have been specifically designed for youth, or are conventional health facilities that have been adapted to serve AYA by providing tailored information and care that is appropriate to the developmental stage [34].

Adolescent responsive mobile clinics that offer integrated health services could ideally link potential patients to conventional clinics, and thus achieve more timeous risk reduction and treatment initiation [33, 34]. Although considered feasible and cost-effective for providing diagnostics to those at-risk of HIV acquisition in difficult to reach areas [24, 35–37], user acceptability of these services for AYA has yet to be evaluated.

The primary objective of this study was to evaluate the acceptability of an AYA friendly mobile SRHS that encourages health-seeking in high disease burden settings in Cape Town, South Africa. The secondary objective was to compare AYA usage and HIV outcomes at the mobile clinic versus that among AYA attending four conventional primary healthcare clinics that operate in the same health district over a similar period.

## Methods

### Study setting and participants

The study was conducted in the Klipfontein Health Sub-structure, a high disease burden, resource limited, densely populated area in the Cape Town Metropolitan, South Africa. The mobile clinic, known as the Tutu Teen Truck, is implemented by the Desmond Tutu HIV Foundation, a non-governmental organisation providing HIV related services and conducting HIV and health related research in Cape Town, South Africa. The mobile clinic provided an adolescent and youth-friendly wellness service where AYA (12–24 years) could access screening for HIV, STIs, tuberculosis (TB), high blood pressure, diabetes mellitus, obesity and pregnancy.

From December 2016 to April 2017, AYA between 16 and 24 years old visiting the Tutu Teen Truck were recruited to participate in an acceptability study through purposive, convenient sampling. The mobile clinic parked in high traffic locations, such as commuter hubs and shopping centres, and offered free screening and testing. These locations were chosen in partnership with community representatives. Additionally, clinic records at the mobile clinic and four conventional clinics were reviewed to compare the characteristics of AYA 12–24 years. The research was approved by the Institutional Review Board (IRB) at the University of Cape Town (HREC Ref 141/2016). In consultation with the UCT IRB, a waiver of parental consent was obtained for the study on the grounds that adolescents access these services without need for parental consent, and the act of obtaining parental consent for the research could create a barrier to participation and undermine the acceptability objective.

### Procedure

Participants that self-presented at the clinic were greeted by a staff member, who recorded their demographic information electronically on a tablet device and linked this information to the participant’s fingerprint on biometric software. After registration, a trained healthcare worker screened and tested participants for HIV, pregnancy, hypertension, diabetes, and obesity using point-of-care diagnostics. Participants were also screened for symptoms of STIs and TB. Participants were not required to have all tests done although the whole spectrum was offered to all where appropriate, and pregnancy checks were offered to women. After completing HCT, participants were invited to join the study. Written consent was obtained from all participants prior to completing the researcher administered questionnaire, which recorded demographics, acceptability of the clinic, and HIV risk perception. In addition, permission was obtained from the Cape Town Department of Health to review clinic records for four conventional government clinics in the same location during the period in which the study ran. Clinics were chosen based on proximity to the area in which the mobile clinic operated.

### Design

This was a cross-sectional acceptability study which compared usage statistics of a mobile clinic versus conventional clinic facilities in the same district. Since no pre-existing acceptability instrument could be obtained, an 11-item scale was developed, which was derived from common desirable aspects of acceptable healthcare services [6]. Participants were asked to rate the 11 aspects of the mobile clinic service by answering Likert-type scale questions with ratings that ranged from 1 to 5. A score of 5 indicated greater acceptability for all items in the scale except for a question that asked whether participants feared being seen at the clinic, which was reverse scored. The sum of the individual scale items (Table 2, Additional file 1) was used to generate an acceptability score. Participants were also asked to rate on a Likert-type scale their risk of three chronic diseases, including diabetes, hypertension and HIV. All analyses were conducted using Stata 14.0 (Stata Corporation LP, College Station, TX). Results were analysed for significant associations with sample demographics. Sample characteristics included age, gender, marital status, education, employment status, whether participants had ever tested, type of dwelling and HIV status. After calculating the proportions of HIV testing diagnoses at the mobile clinic and the conventional clinics, significant differences in proportions were analysed. Bivariate regression analyses were used to identify statistically significant associations and all significant associations (*p* = 0,05) were included in the multivariate regression model. Associations that retained significance were retained in the model.

## Results

A total sample of 303 (19% male) mostly Xhosa speaking (93%) participants between the ages of 16 and 24 years (mean age 19,7) were retained in the analysis (Table 1). Most participants were unemployed (86%) and did not earn an income (83%). Half (*n* = 150, 50%) of the participants lived in informal housing and most (93%) had previously tested for HIV.

**Table 1:** Participant demographics and bivariate regressions for acceptability of the mobile clinic

	n (%)	mean^a^	p^b^
Total	303 (100)	4.6	
Age (*m* = 19,7)	303 (100)		0.394
Sex			0.341
Female	246 (81.2)	4.6	
Male	57 (18.8)	4.6	
Employment			0.147
Unemployed	259 (85.5)	4.6	
Employed	44 (14.5)	4.5	
Income			0.014**
Income	53 (17.5)	4.5	
No Income	250 (82.5)	4.6	
Education			0.007**
Primary School	3 (1)	4.4	
High School	230 (75.9)	4.6	
College/ University	70 (23.1)	4.7	
Dwelling type			0.590
Formal housing	153 (50.5)	4.6	
Informal housing	150 (49.5)	4.6	
Marital Status			0.515
Single	287 (94.7)	4.6	
Cohabiting	13 (4.3)	4.7	
Married	3 (1)	4.6	
Ever Tested			0.001**
Never Tested	22 (7.3)	4.4	
Tested Before	281 (92.7)	4.6	
HIV Status			0.345
HIV positive (new diagnosis)	8 (3.3)	4.7	
HIV negative	232 (96.7)	4.6	

** *p* < 0.01

^a^Mean Acceptability scores

^b^Bivariate regression for Acceptability

When asked if they had used healthcare services in the past, most participants reported that they had used hospital services (47%), followed by clinics (31%) and mobile clinics (22%). No adverse experiences were reported on the mobile clinic.

Since this was a newly created instrument for measuring acceptability, a Cronbach’s alpha test of reliability was conducted and indicated acceptable internal consistency (α = 0,77). All surveyed participants were either happy, or very happy with the duration of the mobile clinic visit (Table 2). Almost all participants (99%, *n* = 301) stated that the mobile clinic staff were friendly or very friendly. When asked if they would tell others about the mobile clinic service, nearly all (99%, *n* = 302) stated that they would. Most participants (96%, *n* = 290) stated that they believed the service was confidential and (92%, *n* = 279) were not concerned that someone they knew would see them at the service. All except one (99%, n = 302) stated they would reuse the service. Most participants (90%, *n* = 273) stated that the mobile service was better or much better than conventional services, while a minority (10%, *n* = 29) stated that services at mobiles and conventional clinics were the same. A single participant (0.3%) stated that conventional clinic services were better or much better. The length of time it took to be seen at a conventional clinic facility was the most often cited (47%, *n* = 143) barrier to accessing healthcare, followed by unfriendly staff (31%, *n* = 93) and distance (22%, *n* = 67). When asked to rate their risk of diabetes, hypertension and HIV infection on a Likert-type scale, 65, 67 and 76% of participants stated that they were not at risk of these conditions respectively. A Wilcoxon signed-rank test showed that risk for HIV was rated significantly lower than either risk for diabetes (Z = -2.898, *p* = 0.004) or hypertension (Z = -3.165, *p* = 0.002).

**Table 2:** Acceptability ratings of the mobile clinic

Acceptability (1 = poor rating, 5 = high rating except where indicated otherwise)	n	Mdn	(IQR)	1		2		3		4		5	
				n	%	n	%	n	%	n	%	n	%
1. How easy was it to understand the counselling at the mobile clinic?	303	4.00	(4–5)	0	0%	1	0%	0	0%	166	55%	136	45%
2. Please rate how helpful was the mobile clinic service?	303	5.00	(4–5)	0	0%	0	0%	1	0%	138	46%	164	54%
3. Would you consider using the mobile clinic service again?	303	5.00	(4–5)	1	0%	0	0%	0	0%	113	37%	189	62%
4. How likely are you to tell others about the mobile clinic service?	303	5.00	(5–5)	1	0%	0	0%	0	0%	2	1%	300	99%
5. How happy were you with the time it took to be seen at the mobile clinic?	303	5.00	(4–5)	0	0%	0	0%	0	0%	95	31%	208	69%
6. Please rate how friendly was the clinic service?	303	5.00	(5–5)	0	0%	2	1%	0	0%	68	22%	233	77%
7. How do mobile clinics compare with traditional clinics/ hospitals?	303	5.00	(4–5)	0	0%	1	0%	29	10%	81	27%	192	63%
8. How do mobile clinics staff compare with traditional clinics/ hospitals?	303	5.00	(4–5)	0	0%	1	0%	27	9%	76	25%	199	66%
9. How concerned are you that someone may see you at the mobile clinic? (reverse scored)	303	5.00	(5–5)	1	0%	19	6%	4	1%	13	4%	266	88%
10. Compared with traditional clinics, mobile clinics are 1 = much worse, 5 = much better	303	5.00	(4–5)	11	4%	29	10%	30	10%	64	21%	169	56%
11. Overall, how would you rate your experience at the mobile clinic?	303	5.00	(4–5)	0	0%	0	0%	14	5%	119	39%	170	56%

In the multivariate analysis (Table 3), higher income (*p* = 0.004, CI −.266–.052), higher education (*p* = 0.003, CI .022 .108) and having been previously tested for HIV (*p* = 0.008, CI .053 .360) remained associated with higher acceptability scores, but there was low predictive value for the effect on acceptability (R-squared = 0.079).

**Table 3:** Multivariate regression analysis model for factors impacting acceptability of the mobile clinic

Usability R2 = 0,079	Estimate	SE	t	p	95% CI
Income	−0,16	0,054	−2,92	0,004	(−0,27; −0,05)
Education	0,06	0,022	2,98	0,003	(0,02; 0,11)
Ever tested	0,21	0,078	2,65	0,008	(0,05; 0,36)
Constant co-efficient	4,19	0,099	42,22	0,000	(4,00; 4,39)

Because utilization of the mobile clinic was voluntary, the characteristics of the AYA who attended the Tutu Teen Truck was compared with all the AYA who attended the four nearest conventional clinic facilities in the area over the same period as a measure of usage. Between December 2016 and April 2017, 4887 patients (Table 4) between the ages of 12 and 25 visited SRHS at the clinic facilities (3737 visited the four conventional clinics, and 1150 visited the adolescent-friendly mobile clinic). The mean age for patients in this age group was 19,1 years (19,3 years at the conventional clinic, 18,4 years at the mobile clinic).

**Table 4:** Comparison of clinic type by age, gender, HIV result and pregnancy result

	Total		Conventional		Mobile		p
	n	%	n	%	n	%	
Overall	4887	100	3737	76,5	1150	23,5	
Age							0,000**
Mean	19,1		19,3		18,4		
Median	19		19		17,9		
12–14	507	10,4	341	9,1	166	14,4	
15–16	680	13,9	439	11,8	241	21	
17–19	991	20,3	699	18,7	292	25,4	
20–25	2709	55,4	2258	60,4	451	39,2	
Sex							0,000**
Female	4090	83,7	3244	86,8	846	73,6	
12–14	397	9,7	289	8,9	108	12,8	
15–16	597	14,6	414	12,8	183	21,6	
17–19	862	21,1	645	19,9	217	25,7	
20–25	2234	54,6	1896	58,4	338	40	
Male	797	16,3	493	13,2	304	26,4	
12–14	110	13,8	52	10,5	58	19,1	
15–16	83	10,4	25	5,1	58	19,1	
17–19	129	16,2	54	11	75	24,7	
20–25	475	59,6	362	73,4	113	37,2	
HIV test result							0,000**
HIV+	119	2,4	73	2	46	4	
12–14	1	0,2	1	0,3	0	0	
15–16	6	0,9	1	0,2	5	2,1	
17–19	16	1,6	6	0,9	10	3,4	
20–25	96	3,5	65	2,9	31	6,9	
Female HIV+	103	2,5	64	2	39	4,6	0,000**
12–14	1	0,3	1	0,3	0	0	
15–16	5	0,8	1	0,2	4	2,2	
17–19	14	1,6	5	0,8	9	4,1	
20–25	83	3,7	57	3	26	7,7	
Male HIV+	16	2	9	1,8	7	2,3	0,642
12–14	0	0	0	0	0	0	
15–16	1	1,2	0	0	1	1,7	
17–19	2	1,6	1	1,9	1	1,3	
20–25	13	2,7	8	2,2	5	4,4	
Pregnancy tests	654		496		158		0,000**
Pregnant	167	25,5	156	31,5	11	7	

Sixteen percent (16,3%) of patients were male, with the mobile clinic recording a higher proportion of males (26,4%) than the conventional clinics (13,2%). The overall HIV prevalence was 2,4%, where prevalence was 2% at the conventional clinics, and 4% at the mobile clinic. The HIV prevalence in the ≥20 year old age group was more than double that of the 17–19 year old group. The univariate analysis showed that patients who visited the mobile clinic differed significantly by age, sex, HIV result and pregnancy status. When HIV test result was disaggregated by age, males at the mobile were not more or less likely to be HIV positive, while females were more likely to test HIV positive at the mobile. At the mobile clinic a quarter (25,7%) of patients reported that this was their first HIV tests (this data is not recorded in the conventional clinic records).

## Discussion

Mobile services offer acceptable, accessible and cost-effective youth tailored services and can provide a gateway into HIV prevention and treatment for adolescents [1]. The results here echo other studies indicating widespread acceptability and uptake of mobile health services in underserved populations [37, 38]. Acceptability was universally high in the sample, with the mobile clinic being perceived as efficient, confidential, friendly, easy to access, with information that was easy to understand. Even though almost all (96%) participants rated the mobile service as confidential, a small number (7%) were concerned that they might be seen at the clinic. Confidentiality and privacy are high priorities and it was exceptional that such a visible clinic was perceived to be confidential and private and that all participants except one were likely or very likely to tell others about the mobile. Such a finding is counterintuitive, but indicates that participants were comfortable accessing the mobile clinic in public spaces.

When the data from the two clinic types were compared, the results showed that the mean age was lower for those accessing the mobile clinic and that the mobile attracted a higher proportion of young men than the conventional clinics. This is an encouraging finding since men generally access conventional facilities at lower rates and are less likely to test and know their status than women. Consequently, mobile clinics may provide opportunities for prevention and treatment to reduce morbidity and mortality amongst young men. Moreover, the mobile clinic found a higher yield of HIV positive young people than the conventional clinics. When disaggregated by sex, HIV prevalence remained significantly higher for women visiting the mobile clinic versus the conventional clinic, but not for men. This may be due to the national trend for a delayed upswing in HIV prevalence for men when compared with women [39]. The pregnancy rates between the two facility types also differed significantly where more young women tested positive for pregnancy at the conventional facilities. This may be an indication that mobiles could be effective sites for implementing convenient family planning counselling and for dispensing contraceptive methods for young people.

It was significant that previously tested participants gave higher ratings of the mobile clinic service. Qualitative data from a study with South African AYA, which investigated young people’s preferences for desirable healthcare services, showed that trust between young people and healthcare providers is vital in supporting youth engagement in care [40]. It may be that those who had previously tested had a comparator and had subsequently formed good impressions of the professional-patient relationships with health providers on the mobile service, and as a result rated the service more highly. Even though debut testers gave lower ratings than repeat testers, their ratings were still high.

Overall, these results resonate with findings from elsewhere in South Africa showing that mobile clinics are an effective strategy for overcoming the barriers to reaching young people, and as an added benefit, are effective at reaching young men who access conventional clinics at lower rates than women [38]. The need for continued awareness raising, community-based efforts for HIV testing and case-finding of young people living with HIV is supported by the finding that participants were more likely to deny vulnerability to HIV than other chronic conditions.

Self-selection for the service and the study was a limitation that may have influenced the high levels of acceptability. Even though the mobile service strategically selected high disease burden communities, patients who know they are high risk, or who presume an HIV positive diagnosis, may avoid diagnostic services because of anticipated stigma or self-stigmatisation around an HIV positive result, denial, or a desire to delay knowing the result. Fine-grained geospatial mapping may help to target areas as specific as streets and blocks to improve the yield of those who are most at risk. Further to this limitation is that questionnaires were researcher-led, and therefore possibly subject to social desirability bias. One way to control for this may be to use computer-assisted or tablet-based self-administered surveys, or community-based surveys away from clinic settings to minimise this bias. Even so, while self-selection may have positively skewed acceptability, the comparison with the conventional clinics showed that the mobile clinic saw proportionately more HIV positive patients, indicating that mobile clinics may be more successful at finding new HIV infections.

The study did not record participation refusals and associated reasons for refusal. The majority (68%) of those who completed the questionnaire were female, and therefore an equal spread of answers across the sexes was not established. However, even though young men only constituted 32% of those who completed the questionnaire, this figure was higher than the proportion of men who visited the mobile clinic during the course of the study (26%). Since inception in 2015, men have constituted 40% of clinic visits at the mobile, which is encouraging. While the higher proportion of young men testing at the mobile may indicate that mobile testing is more desirable to men than testing at a conventional facility, the proportion of those HIV positive was not significantly different from the conventional clinic. Future research could investigate mobile clinics that incorporate young men’s healthcare needs since dedicated men’s clinics may be a more attractive option because they are tailored to men.

## Conclusions

Mobile clinics can offer convenient and complimentary service to conventional clinics and the ability to actively target at-risk communities can provide earlier detection of HIV and provide relief to brick and mortar clinics. Additionally, mobile SRHS can make health-seeking more convenient for AYA. Examples of possible models for mobile units include diagnostic versus one-stop mobile clinics that provide a range of diagnostic, prevention and treatment services. Cost effectiveness of an integrated, one-stop diagnostic and prevention and treatment service should be determined, but may go some way towards addressing attrition between testing and treatment services [41]. Mobile clinics can target high risk communities and can provide convenient access to much needed SRHS. This delivery platform leans heavily into decreasing effort, decreasing time, improving the emotional experience, and ensuring easier uptake for at-risk AYA by strategically locating the service where it is needed.

This study demonstrated that mobile clinics are more desirable and can achieve high acceptability amongst young people in need of sexual and reproductive healthcare in a resource-limited, high disease burden setting in South Africa. The results further demonstrated that this mobile clinic offered convenience, trusting relationship and confidentiality. Accordingly, mobile clinics should be used as part of a multi-faceted approach to increase the convenience of HIV testing and counselling for adolescents and young adults in resource-limited settings. Future studies to evaluate acceptability of these services amongst those even harder to reach, including young men and boys and debut testers are warranted.

## Additional file

Additional file 1 Acceptability questionnaire. (PDF 62 kb)

## Data Availability

The datasets used and/or analysed during the current study are available from the lead author on reasonable request.

## Abbreviations

ART: Antiretroviral treatment
AYA: Adolescent and young adult
HIV: Human immunodeficiency virus
PrEP: Pre-exposure prophylaxis
SRHS: Sexual and reproductive health services
STIs: Sexually transmitted infections
TB: Tuberculosis
