# Supplementary material for: Mobile sexual health services for adolescents: investigating the acceptability of youth-directed mobile clinic services in Cape Town, South Africa
Source: BMC Health Serv Res. 2019 Aug 19;19:584. doi: 10.1186/s12913-019-4423-4 (PMC6701080; doi:10.1186/s12913-019-4423-4)
Supplement: Supplementary file 1 — Acceptability questionnaire. (PDF 62 kb) [file 12913_2019_4423_MOESM1_ESM.pdf]

Mobile Services: feasibility and acceptability questionnaire

A. Referred for
   1. HIV care   2. ART care   3.TB symptoms   4. Diabetes   5. HPT   6. STI   7. None   8. Other

B. Interview details
   1. Date of test (dd/mmm/yyyy) ____/________/________
   2. Date of birth (dd/mmm/yyyy) ____/________/________
   3. Name of counsellor _______________________
   4. Demographics
      a. Nationality _______________________
   5. Language: 1: Afrikaans   2: English   3: Xhosa   4: Other_______________________

Greeting: Thank you for using the test, we really appreciate that you agreed to take part in this research! We would like to know what it was like to use the mobile clinic.

1. Before testing with the Tutu Tester, had you ever had an HIV test?
Yes   No   Unsure

2. Please circle which services you have accessed?
Mobile clinic   Clinic   Hospital   Private doctor   Other: _______________________

3. Mobile Clinic survey

1. On a scale of 1 to 5, how easy was it to understand the counselling at the mobile clinic?
1 not at all easy   2 not easy   3   4 easy   5 very easy

2. On a scale of 1 to 5, please rate how helpful was the mobile clinic service? 1 = not at all helpful, 5 = very helpful
1 not at all   2 not helpful   3   4 helpful   5 very helpful

3. On a scale of 1 to 5, would you consider using the mobile clinic service again? 1 = not at all, 5 = very much
1 not at all   2 no   3   4 yes   5 very much

4. How likely are you to tell others about the mobile clinic service? 1 = never, 5 = very likely
1 not at all   2 not likely   3   4 likely   5 very likely

5. How happy were you with the time it took to be seen at the mobile clinic?
1 very unhappy   2 unhappy   3   4 happy   5 very happy

6. On a scale of 1 to 5, please rate how friendly was the clinic service?
1 very unfriendly   2 unfriendly   3   4 friendly   5 very friendly

7. How confidential is the mobile clinic service? (Your information will be kept private?)
1 not confidential   2 don't know   3 Confidential

8. How do mobile clinics compare with traditional clinics/ hospitals?
1 much worse   2 worse   3 same   4 better   5 much better

9. How do mobile clinics staff compare with traditional clinics/ hospitals?
1 much worse   2 worse   3 same   4 better   5 much better

10. Which health facilities have the best healthcare experience?
1 Mobile Clinics are better   2 Service is about the same   3 Clinic facilities are better

11. How concerned are you that someone may see you at the mobile clinic?
1 not at all   2 not really   3 don't know   4 yes   5 very much

12. Compared with traditional clinics, mobile clinics are
1 much worse   2 worse   3 same   4 better   5 much better

13. Overall, how would you rate your experience at the mobile clinic?
1 very bad   2 bad   3 okay   4 good   5 very good

14. How at risk are you for diabetes?
1 not at all   2 not really   3 don't know   4 yes   5 very much

15. How at risk are you for high blood pressure?
1 not at all   2 not really   3 don't know   4 yes   5 very much

16. How at risk are you of contracting HIV?
1 not at all   2 not really   3 don't know   4 yes   5 very much

17. What makes it difficult to access healthcare? (Do not prompt)
a. Too far
b. Unfriendly staff
c. It takes too long
d. Other_______________________

18. Have you experienced any bad consequences because of the mobile service?
Yes   No

19. **If yes for 18, what were the bad consequences? (circle 1 letter)**
a. I did not enjoy the experience
b. It was distressing
c. I fear someone might find out I tested
d. Other_______________________

20. Demographics (circle relevant answer/s)

1. Marital status	Single	Cohabiting	Married	Divorced
2. Sex	Female	Male		
3. Employed	Yes	No		
4. Monthly income	Yes	No		
5. Highest education level (circle 1)				
a. primary school	1 Some	2 Complete		
b. High school	3 Some	4 Complete		
c. University	5 Some	6 Complete		
6. Type of dwelling	Formal	Informal		

Greeting: Thank you so much for taking part in our research, we really appreciate your time!
